# Knowledge regarding breast self-examination among the women in Nepal: A meta-analysis

**DOI:** 10.3126/nje.v9i2.24684

**Published:** 2019-06-30

**Authors:** Brijesh Sathian, Mohammad Asim, Ahammed Mekkodathil, Sruthi James, Angela Mancha, Arnab Ghosh

**Affiliations:** 1 Academic Research Associate, Clinical Research, Trauma and Vascular Surgery, Surgery Department, Hamad General Hospital, Doha, Qatar.; 2 Injury prevention coordinator, Clinical Research, Trauma and Vascular Surgery, Surgery Department, Hamad General Hospital, Doha, Qatar.; 3 Research Coordinator, Clinical Research, Trauma Surgery, Surgery, Hamad General Hospital, Doha, Qatar.; 4 Professor, Department of Pathology, Manipal College of Medical Sciences, Pokhara, Nepal.

**Keywords:** Breast self-examination, cancer, women, knowledge, Nepal

## Abstract

**Background:**

Screening interventions for the early diagnosis of breast cancer are associated with better clinical outcomes. Developing nations such as Nepal reportedly have lesser frequency of female university graduates (UGs) and therefore public awareness and education remains central in the early diagnosis of breast cancer. The current meta-analysis was aimed to assess the knowledge about breast self-examination (BSE) among women of Nepal.

**Materials and Methods:**

We have conducted the literature search using electronic databases such as PubMed, MEDLINE, Cochrane library and Google scholar. The search terms utilized were “breast self-examination”; “knowledge”, “awareness”; and “attitude” in various combinations AND “Nepal” in the title or abstract. Additional searches were conducted with the help of cross references quoted in the selected studies and review articles. Data were retrieved using excel sheets which were pilot tested. Data were independently abstracted by the four authors using a standardized data collection form. Findings from the various studies were pooled together for the sake of analysis, if appropriate.

**Results:**

The search yielded 36 articles; 27 duplicates and review articles were excluded and a further 4 articles not relevant were excluded. Finally, 5 original studies met the inclusion criteria. Total pooled sample size for assessing knowledge was 1910. The overall pooled knowledge about breast self-examination was found to be 27% with a 95% CI [23-31].

**Conclusion:**

The pooled estimates demonstrated that the overall knowledge of breast self-examination was inadequate among women in Nepal. Therefore, prompt capacity building measures are warranted to enhance the public awareness towards BSE.

## Introduction

Breast cancer remains the third most frequent incident malignancy among females worldwide, with reported incident cases of 1.7 million (95% UI, 1.6-1.8 million) in the year 2016 [1]. It is the major cause of cancer related morbidity and mortality with an estimated 535 000 deaths and 14.9 million disability-adjusted life years (DALYs) [1].

Screening interventions for the early diagnosis of breast cancer are associated with better clinical outcomes. Developing nations such as Nepal reportedly have lesser frequency of female university graduates (UGs) and so public awareness and education plays a vital role in the breast cancer diagnosis during early phase. It has been identified that the vast majority of females in the developing nations are unaware about performing regular breast self-examination (BSE) and thus have a lower rate of BSE. This could be attributed to the fact that women in the developing countries are afraid of finding that they have breast cancer, inappropriate knowledge in performing BSE and ignorance about the measures if a lump is identified. Earlier studies have demonstrated that such barriers about knowledge and attitude can be addressed by appropriate education on BSE [2-10].

Mammography, clinical breast examination and BSE are the most widely used modalities of screening for early diagnosis of breast cancer which contributes to the prognosis and therefore decreased mortality rates. Although these breast cancer screening modalities have certain pros and cons; but is still considered for early detection. Therefore, the goal of the healthcare system is to promote awareness among women to identify signs and symptoms of breast cancer. The current meta-analysis was aimed to assess the knowledge about BSE among women in Nepal.

## Methodology

The current meta-analysis was performed and reported as per the Preferred Reporting Items for Systematic Reviews and Meta-Analyses (PRISMA) Statement and prescribed guidelines.

### Literature searches

We have conducted the literature search using electronic databases such as PubMed, MEDLINE, Cochrane library, and Google scholar. The search terms utilized were “breast self-examination”; “knowledge”, “awareness”; and “attitude” in various combinations AND “Nepal” in the title or abstract. Additional searches were conducted with the help of cross references quoted in the selected studies and review articles.

### Inclusion and exclusion criteria

The present meta-analysis included articles based on (1) originality, (2) English language; (3) period of publication (01 January 2000 till 31st January 2019); (4) full-text available; (5) assessment of knowledge of BSE; (6) study population was from Nepal and (7) study population of females of any specified age. We have excluded related publications which are not original studies i.e. narrative reviews, letters to the editor, commentaries and only published abstracts.

The appropriate inclusion and exclusion criteria were considered depending upon the fact that whether the study was based on the information about knowledge of BSE in Nepal, regardless of the type of study population. Therefore, we have included even studies with smaller sample sizes for the analysis. The Mesh terms employed during the literature search process include “Breast Self-Examination”; “Knowledge, Attitudes, Practice”; “Awareness” and “Nepal”.

### Data extraction

Initially, the study titles resulted from the searches in different databases were screened in order to select the relevant publications. Then critical review of the abstracts and full texts was performed according to the inclusion and exclusion criteria for final selection of articles. Data (abstracts and full-text articles) were independently reviewed and abstracted by the three researchers (BS, MA, & AM) using a standardized data collection form. The collected data included information of authors, study origin, targeted population, study setting and duration, selection criteria (inclusion or exclusion), sources of data and assessment measures, sample size estimation, distribution of age, and awareness status.

### Methodological quality

The methodological quality of the chosen articles was evaluated based on five STROBE criteria from the given checklist such as design of the study, study settings, study participants, source of data/assessment and sample size of the study. The STROBE checklist and the selected five criteria from the checklist were most appropriate for the methodological quality assessment of the epidemiological observational studies.

### Data Analysis and Synthesis

Descriptive statistics and 95% Confidence Interval [CI] were used to summarize knowledge percentage estimated from individual studies. The findings of the statistical tests for heterogeneity help us to decide for the consideration of either fixed effect or random effects model. Moreover, the Cochrane Q homogeneity test was performed to assess the data heterogeneity and the finding was significant, if p value was less than 0.05. Notably, the fixed effect model was considered, if the studies were found to have statistical homogeneity. On the other hand, a random effects model was utilized, if studies had statistical heterogeneity. The I^2^ test is the ratio of true heterogeneity to the total variation in the observed effects. The interpretation of I^2^ test was roughly represented as percentages which ranges from 0 to 25% (might not be important); 25 to 50% (may represent moderate heterogeneity); 50 to 75% (may represent substantial heterogeneity); and more than 75% (considerable heterogeneity). Funnel plot and Doi plot were used to find out the publication bias. Pooled estimates were calculated using R 3.5.1 software.

## Results

**Figure 1:**
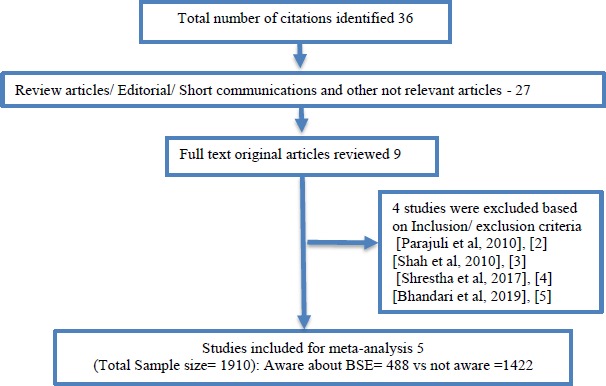
Flow diagram of study selection process for systematic review.

The search in PubMed, Medline, Cochrane library, Google scholar and consideration of cross references yielded a total of 36 articles; of which 27 duplicate studies identified by different databases and review articles were excluded. Moreover, based on the search for relevant titles and/or abstracts which were evaluated in detail, further 4 articles [2-5] were excluded from the analysis. Finally, a total 5 original studies [6-10] fulfill all the inclusion criteria and were included in the analysis (Figure 1 & Table 1).

Table 1 demonstrated the summary and quality assessment of the selected studies for the meta-analysis in the current review [6-10]. Among the 5 included studies, knowledge was measured by administering a questionnaire which addresses various aspects of breast self-examination [6-10]. Three studies were conducted among general population [8-10]; one study was performed in hospitalized women and the other one was based on women visiting the hospital [6, 7].

**Figure 2:**
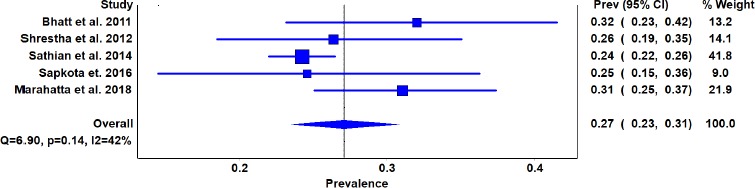
Forest plot about the knowledge of breast self-examination

Total pooled sample size for assessing knowledge was 1910. Figure 2 show the pooled overall knowledge regarding breast self-examination was 27% with a 95% CI [23, 31].

### Heterogeneity among included studies

For this meta-analysis, the findings for the test of heterogeneity regarding the knowledge of breast self-examination are demonstrated towards the bottom of the forest plot in the line: for all Q [χ2] = 6.9, P=0.14, I^2^=42% (Figure 2), However, due to the fact that the I^2^ was found to be >25%, a random effect model was considered for this meta-analysis. Tau^2^ reflects the amount of true heterogeneity among the studies, which was less in our study (tau^2^=0.004).

### Publication bias and funnel and Doi plots

**Figure 3:**
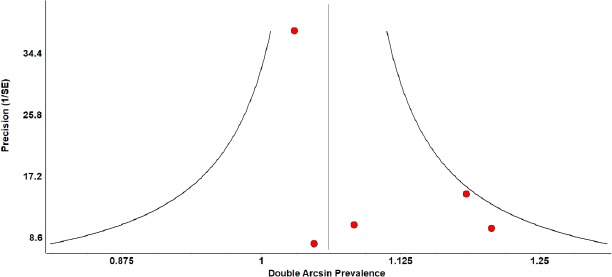
Funnel plot about the knowledge of breast self-examination

**Figure 4:**
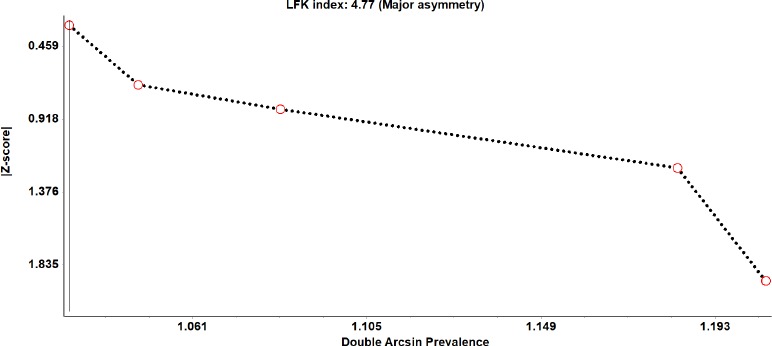
Doi plot about the knowledge of breast self-examination

With respect to all above mentioned tests, the sensitivity analysis provided consistent results. With the help of a visual inspection of the funnel plot and Doi plot for the included studies, there seems to be no evidence of publication bias (Figure 3 and 4). The publication bias was ascertained by the Doi plot which demonstrated major asymmetry by the asymmetry index (LFK index = 4.77).

## Discussion

To the best of our knowledge, the present meta-analysis is a primary effort for the assessment of the knowledge regarding BSE among women in Nepal. This meta-analysis was based on five non-randomized control trials conducted to assess and explore the knowledge of BSE, and we found that the knowledge on BSE among women in Nepal was poor (27%). We have observed variability in the type and amount of information included in each selected study; however, we believe that this variability will not affect the pooled results since the outcomes of these evaluated studies were homogenous.

### Socio-demographic characteristics of the studies

Table 2 shows that majority of the participants belonged to Hindu religion (78.7% to 96.7%) [6, 8-10] of which Brahmin/ Chettri was the predominant caste (25.1% to 55.7%) [7-10]. An earlier study from south Africa showed that Hindu women of Indian origin do not believe themselves to be at higher risk of developing breast cancer than the average woman [11]. These women also showed poor BSE practice attribute to the absence of neither strong positive nor negative perception of BSE [11] which could be applicable to the religious belief of Hindu women in Nepal as well.

Also, higher frequency of respondents was educated to senior secondary school level or were college graduates which are more likely to be aware about BSE [6, 8, 10]. Our findings are contrary with an earlier study which showed that the educational level of respondents is not associated with the knowledge of BSE [12]. The respondents were more likely to be housewives (11.3% to 58.2%) [6-8].

### Knowledge of warning signs of breast cancer

Knowledge regarding various warning signs of breast cancer can be life saving for women. In our analysis breast lump (varied 4.6% to 89%), lump under armpit (ranges from 4.8% to 100%) and bleeding or discharge from nipple (4.4% to 21.2%) were the frequent warning signs of which women are aware (Table 3). Our finding is consistent with an earlier study which reported frequent warning signs of breast cancer to be nipple discharge, painless breast lump and changes in the skin of the breast [13].

To date, the efforts for cancer prevention remains less effective for most prevalent cancers such as breast cancer [1]. Early detection and management remain crucial, however, even in the best-case scenario for early cancer detection only a fraction of cancers can be preventable, despite the fact that delivery of universal health care access is of paramount importance for cancer control [14]. Our finding about poor knowledge on BSE among women in Nepal will ultimately contribute to the knowledge base for developing prevention strategies for breast cancer in Nepal.

### Breast self-examination as an alternative to mammography

Awareness regarding mammogram was found to vary from 8.2% to 19.9% in the current analysis (Table 3). Hackshaw et al. reported in their meta-analysis that BSE is primarily suggested for prevention of breast cancer [15]. Contrarily, despite a clear documentation and evidence suggested by contemporary studies that mammography screening is ineffective in decreasing the mortality from breast cancer [16-18]. Therefore, BSE may be considered as an effective alternative measure for early diagnosis and better treatment.

### Limitation of the study:

This meta-analysis included a total pooled sample size of 1910 for assessing knowledge about BSE. One of the limitations in the study was all the questionnaires were not validated properly with reliability analysis. Differences in questionnaires, procedure of data collection and assessments, heterogeneity between the studies and the quality of data sources still considered as major challenges as well as over-reporting of BSE knowledge in low-resource settings.

## Conclusion

The pooled estimates demonstrated that the overall knowledge of BSE was inadequate among women in Nepal. Therefore, prompt capacity building measures are warranted to enhance the public awareness towards BSE.

### Future scope of the study:

More studies based on a validated questionnaire on BSE is warranted.

### What is already known on this topic:

Previous studies conducted based on small sample sizes from different areas of Nepal revealed variability in knowledge of BSE among women in Nepal.

### What this study adds:

This study found the pooled estimate for BSE knowledge among women in Nepal.

## Figures and Tables

**Table 1:** summary and quality assessment of the eligible studies for the meta-analysis in the current review

Study name	Sample size	Origin	Study Duration	Age	Study population and design	Sampling	Questionnaire	Findings	STROBE
**Bhatt et al. 2011 [6]**	100	Tribhuvan	1 year and 2 months	Mean 37 years	Women admitted in hospital, Cross sectional	Systematic Sampling	Physician administered questionnaire, validated by pre-test	BSE Knowledge = 32%	Complete
**Shrestha et al. 2012 [7]**	110	Lalitpur	4 weeks	20-60 years	Women visiting the hospital, Cross sectional	Purposive Sampling	Structured questionnaire, validated by subject experts only	BSE Knowledge = 26.4%	Complete
**Sathian et al. 2014 [8]**	1420	Pokhara	6 months	15-68 years	Female, Cross sectional	Convenient sampling	Semi structured questionnaire, validated by piolet study	BSE Knowledge = 24.2%	Complete
**Sapkota et. al 2016 [9]**	61	Biratnagar	-	Mean 16.62 years	Female, Quasi experimental	Random sampling	Semi structured questionnaire, validated by pre-test study	BSE Knowledge = 24.6%	Complete
**Marahatta et al. 2018 [10]**	219	Butwal	6 months	Mean 31.84 years	Female, Cross sectional	Two stage cluster random sampling	Semi structured questionnaire, validated by pre-test	BSE Knowledge = 31.1%	Complete

**Table 2:** Socio-demographic distribution of study participants

Socio-Demographic Variables	Bhatt et al. 2011 [6]	Shrestha et al. 2012 [7]	Sathian et al. 2014 [8]	Sapkota et al. 2016 [9]	Marahatta et al. 2018 [10]
**Religion**					
Hindu	83%	-	78.70%	96.70%	82.60%
Muslim	3%	-	3.30%	-	3.20%
Christian	1%	-	3.20%	-	8.20%
Buddhist	11%	-	14.80%	3.30%	-
**Caste**					
Brahmin / Chettri	-	33%	25.10%	55.70%	40.60%
Newar	-	-	7.00%	-	-
Gurung	-	-	8.90%	-	-
Dalit	-	-	24.70%	-	13.20%
Magar, pun, lama	-	-	5.80%	-	-
Janajati	-	-	-	-	33.80%
Madhesi	-	-	-	-	6.80%
Others	-	-	8.40%	44.30%	2.30%
**Education**					
Illiterate	18%	-	8.60%	-	9.60%
Primary	13%	38%	26.00%	-	29.20%
Class 11	-	-	-	29.50%	
Class 12	35%	-	36.10%	70.50%	23.30%
Graduate	32%	-	29.30%	-	37.90%
**Occupation**					
Bank employee	-	-	4.20%	-	-
Chemist	-	-	1.40%	-	-
College Teacher	-	-	2.10%	-	-
Computer Technician	-	-	1.40%	-	-
Cook	-	-	4.20%	-	-
Farmer	-	-	2.00%	-	-
Housemaid	-	-	2.30%	-	-
Housewife	48%	58.20%	11.30%	-	44%
Librarian	-	-	0.90%	-	-
Primary school teacher	-	-	2.60%	-	-
Restaurant owner	-	-	1.40%	-	-
Shopkeeper	-	-	14.10%	-	-
Staff nurse	-	-	4.20%	-	-
Student	6%	-	33.80%	-	19%
Sweeper	-	-	6.60%	-	-
Vegetable vendor	-	-	4.20%	-	-
Waitress	-	-	3.20%	-	-
Others	11%	-	-	-	37%
Teacher	12%	-	-	-	-
Self-Employed	20%	-	-	-	-

**Table 3:** Knowledge regarding breast cancer warning signs, BSE and mammogram in different studies

Warning Signs	Bhatt et al. 2011 [6]	Shrestha et al. 2012 [7]	Sathian et al. 2014 [8]	Sapkota et. al 2016 [9]	Marahatta et al. 2018 [10]
Breast lump	89%	30%	4.60%	-	-
Lump under armpit	-	100%	4.80%	-	-
Bleeding or discharge from nipple	-	21.20%	4.40%	-	-
Pulling of the nipple	-	-	4.60%	-	-
Changes in the position of the nipple	-	-	4.90%	-	-
Nipple rash	-	-	4.50%	-	-
Redness of the breast skin	-	-	4.60%	-	-
Changes in the breast or nipple size	-	-	4.60%	-	-
Changes in the shape of breast or nipple	-	-	4.60%	-	-
Pain in the breast or armpit	-	-	4.80%	-	-
Dimpling of the breast skin	-	9%	4.80%	-	-
BSE Awareness	32%	26%	24.20%	25%	31.10%
Family History of Breast cancer	49%	-	19.90%	-	-
BSE in the last year	-	-	4.30%	-	19.20%
Painless lump	39%	61%	-	-	-
Mammogram	-	8.20%	19.90%	-	-

## References

[1] Global Burden of Disease Cancer Collaboration Fitzmaurice C Akinyemiju TF Al Lami FH Alam T Alizadeh-Navaei R Global, Regional, and National Cancer Incidence, Mortality, Years of Life Lost, Years Lived With Disability, and Disability-Adjusted Life-Years for 29 Cancer Groups, 1990 to 2016: A Systematic Analysis for the Global Burden of Disease Study. JAMA Oncol. 2018 Nov 1;4(11):1553-1568. 10.1200/JCO.2018.36.15_suppl.1568 PMid:29860482; PMCid:PMC6248091. 29860482 PMC6248091

[2] Parajuli P Mandal G. Knowledge about Breast Cancer and Breast Self Examination Practices among Medical, Dental and B.Sc Nursing Students of BPKIHS. Health Renaissance,. 2010;8(3):166-8. 10.3126/hren.v8i3.4209

[3] Shah T Pokharel N Shah S Rai M. Breast and Cervical Cancer Risk Factors and Screening Awareness among Nurses working in Government Sectors in Eastern Region of Nepal. [Online June 2010], [Cited on May 2019] Available from: http://library.nhrc.gov.np:8080/nhrc/handle/123456789/61

[4] Shrestha S Chhetri S Napit J. Awareness on Breast Self Examination among Reproductive Age Women. JCMSN. 2017;13(4):425-9. 10.3126/jcmsn.v13i4.18731

[5] Bhandari PM Thapa K Dhakal S Bhochhibhoya S Deuja R Acharya P Mishra SR Breast cancer literacy among higher secondary students: results from a cross-sectional study in Western Nepal. BMC Cancer. 2016 Feb 18;16:119 10.1186/s12885-016-2166-8 PMid:26887650; PMCid:PMC4758038. 26887650 PMC4758038

[6] Bhatt V Wetz R Shrestha R Shrestha B Shah N Sayami P Breast cancer knowledge, attitudes and practices among Nepalese women. European Journal of Cancer Care 2011; 20: 810-817. 10.1111/j.1365-2354.2011.01272.x PMid:21831258 21831258

[7] Shrestha K Breast cancer knowledge and screening practice among women visited to KIST medical college. Nepal Med Coll J. 2012 Dec;14(4):308-11. 24579540

[8] Sathian B Nagaraja SB Banerjee I Sreedharan J De A Roy B Awareness of breast cancer warning signs and screening methods among female residents of Pokhara valley, Nepal. Asian Pac J Cancer Prev. 2014;15(11):4723-6. PubMed PMID: 24969910. 24969910 10.7314/apjcp.2014.15.11.4723

[9] Sapkota D Parajuli P Kafle TK Effectiveness of Educational Intervention Programme on Knowledge Regarding Breast Self Examination among Higher Secondary School Girls of Biratnagar. BJHS 2016; 1(1): 13-19. 10.3126/bjhs.v1i1.17091

[10] Marahatta S Sharma S. Knowledge and practice of breast self examination among women of reproductive age in Butwal Sub Metropolitan City. Journal of Manmohan Memorial Institute of Health Sciences 2018; 4(1):117-129. 10.3126/jmmihs.v4i1.21149

[11] Govender C Soma P Persad L Moodley J Rajah V. Breast Cancer Health Beliefs and Perceived Barriers to Self-Examination Amongst Hindu Women in South Africa. Journal of Psychology in Africa 2013; 23(1): 101-104. 10.1080/14330237.2013.10820600

[12] Varghese SD Nayak M. Awareness and Impact of Education on Breast Self-Examination Among College Going Girls. Indian J Palliat Care 2011 May-Aug; 17(2): 150-154. 10.4103/0973-1075.84538 PMid:21976857 PMCid:PMC3183606 21976857 PMC3183606

[13] Radi SM Breast cancer awareness among Saudi females in Jeddah. Asian Pac J Cancer Prev, 2013;14, 4307-12. 10.7314/APJCP.2013.14.7.4307 PMid:23991994 23991994

[14] GBD 2016 Risk Factors Collaborators. Global, regional, and national comparative risk assessment of 84 behavioural, environmental and occupational, and metabolic risks or clusters of risks, 1990-2016: a systematic analysis for the Global Burden of Disease Study 2016. Lancet. 2017;390(10100): 1345-1422. 10.1016/S0140-6736(17)32366-8 PMid:28919119; PMCid:PMC5614451. 28919119 PMC5614451

[15] Hackshaw AK Paul EA Breast self-examination and death from breast cancer: a meta-analysis. Br J Cancer. 2003 Apr 7;88(7):1047-53. 10.1038/sj.bjc.6600847 PMid:12671703 PMCid:PMC2376382 12671703 PMC2376382

[16] Nystrom L Larsson LG Wall S. An overview of the Swedish randomised mammography trials: total mortality pattern and the representivity of the study cohorts. J Med Screening 1996; 3: 85-87. 10.1177/096914139600300208 PMid:8849766 8849766

[17] Olsen O Gotzsche PC Cochrane review on screening for breast cancer with mammography. Lancet 2001; 358: 1340-1342. 10.1016/S0140-6736(01)06449-2 11684218

[18] Olsen O Gotzsche PC Is screening for breast cancer with mammography justifiable? Lancet 2000; 355: 129-134. 10.1016/S0140-6736(99)06065-1 10675181

